# Species variation in the effects of dewatering treatment on macroalgae

**DOI:** 10.1007/s10811-018-1420-7

**Published:** 2018-02-20

**Authors:** Joe A. Gallagher, Lesley B. Turner, Jessica M. M. Adams, Sara Barrento, Philip W. Dyer, Michael K. Theodorou

**Affiliations:** 1 Biorefining Group, Institute of Biological, Environmental and Rural Sciences, Aberystwyth University, Gogerddan, Aberystwyth, Ceredigion SY23 3EE UK; 2 Centre for Sustainable Aquatic Research (CSAR), Swansea University, Singleton Park, Swansea, SA2 8PP UK; 3 CIIMAR, CIIMAR–Centre of Marine and Environmental Research, Terminal de Cruzeiros do Porto de Leixões, Av. General Norton de Matos s/n, 4450-208 Matosinhos, Portugal; 4 Centre for Sustainable Chemical Processes, Department of Chemistry, Durham University, South Road, Durham, DH1 3LE UK; 5 Agricultural Centre for Sustainable Energy Systems, Harper Adams University, Newport, Shropshire TF10 8NB UK

**Keywords:** Biorefining-feedstock preservation, Dulse, Ensiling, Kelp, Seasonal variation, Seaweed, Silage effluent production/reduction

## Abstract

Seaweeds can be a valuable resource for biorefinery and biotechnology applications, but their high water content is a recurrent problem and one of the key bottlenecks for their sustainable use. Treatments to increase dry matter content of the kelp *Laminaria digitata* were recently described by the authors. However macroalgae are an extremely diverse group of organisms and compositional variation between species may influence the effects of particular treatments. In this study, potential dewatering treatments including drying, osmotic media, and the application of both organic and mineral acids all followed by screw-pressing have been tested on two other species of kelp (*Laminaria hyperborea* and *Saccharina latissima*) and a red seaweed (*Palmaria palmata*). Conditions that dewatered these species were identified and the data have been combined with the previous results for *L. digitata*. There were significant differences between species across all the traits of interest. However dewatering was highly dependent on specific interactions with both treatment and season of collection. Nevertheless, the dry matter content of brown seaweeds was widely and successfully increased by air drying or acid treatment followed by screw-pressing. The results for *P. palmata* were quite different, particularly with regard to juice production. For this species, acid treatment did not result in dewatering, but dry matter content could be increased by screw-pressing immediately after harvest. Together the data presented here demonstrate that dewatering pre-treatments need to be specific for the type of seaweed to be processed; important knowledge for the future use of this sustainable biomass resource.

## Introduction

Although macroalgae are a valuable food resource in Asia, in the western world, they have traditionally been used mainly for the extraction of chemicals including hydrocolloids (e.g. alginates) and minerals. Many processes have concentrated on obtaining single products but macroalgae have the potential to provide a range of products and by-products, some with high value (Jiang et al. 2016). With the expansion of the biorefining and biotechnological sectors over the last decade, the value of macroalgal biomass for an increasing range of new applications and markets has been recognised. Macroalgae have several advantages over land biomass crops such as sugar cane, soya and corn, as not only do they not require fresh water, agricultural land, fertilizers and pesticides (Adams et al. 2017) but their cultivation can also be used to provide valuable ecosystem services (Buschmann et al. 2017; Chung et al. 2017; Gajaria et al. 2017; Raven 2017) such as bioremediation, carbon sequestration and mitigation of ocean acidification. Furthermore, the appeal of this biomass can be directly attributed to the fact that seaweeds have high productivity, fast growth rates and high polysaccharide content (Loureiro et al. 2015; Suutari et al. 2015). Indeed, the supply of macroalgae for processing has grown steadily and in the past 14 years production has nearly tripled—from 9.3 million tonnes in 2000 to 27.3 million tonnes in 2014 (FAO 2017). Currently, global production is concentrated in Asia (96%) but this will become more widespread as the West starts to exploit local seaweed resources. However, much research is needed to fully exploit the potential of seaweeds in recently identified biotechnological applications (Milledge et al. 2014; Loureiro et al. 2015; Suutari et al. 2015). The sustainable use of macroalgal biomass requires careful management of the biomass-value chain from production (fisheries or aquaculture) through harvesting (mechanical or manual), processing (dewatering, extraction of chemicals, ensiling) and packaging (vacuum, modified atmosphere packaging, ensiling in bulk bags) to transport (cold chain by air, sea or land) and storage. Additionally, there are further challenges that are only now beginning to be addressed by western countries including sporophyte supply, genetic diversity and strain selection, the need for year-round biomass supply and quality control (e.g. heavy metal content).

For biorefinery and biotechnological applications that utilise macroalgae, the most crucial process is dewatering. Macroalgae, like microalgae and most green plants, have water contents typically in the range 74–89% (Adams et al. 2011a; Herrmann et al. 2015; Suutari et al. 2015). Wet biomass is heavy, bulky and costly to transport, deteriorates rapidly and can produce copious quantities of seepage effluent. At least partial drying is desirable before transportation on weight grounds, as well as for prevention of biomass deterioration (Seghetta et al. 2017). Dewatering at an early stage in processing provides better quality material and decreases both transport costs and associated greenhouse gas emissions. However, a sustainable alternative to traditional drying methods, which typically consume fossil energy, is desirable especially when dry feedstock is required for applications such as sustainable fuel production (Milledge et al. 2014, 2015; Herrmann et al. 2015; Song et al. 2015; Milledge and Harvey 2016a; Soomro et al. 2016). Ensiling has been demonstrated to be an effective, low-energy-loss method of preserving seaweed to establish a non-seasonal supply (Herrmann et al. 2015; Milledge and Harvey 2016b). However, ensiling potentially creates effluent as water leaches from the biomass. Effluent production from terrestrial material is commonly controlled through increasing dry matter (DM) content to 25–30% by wilting cut crops in the field prior to ensiling. This results in feed that generally has around 15% lower water content than fresh forage (Haigh 1994; Wright et al. 2000). Thus dewatering can be a useful component of the ensiling process. However reductions of only 1–2% water content between fresh and ensiled macroalgal biomass have been reported for several trials although effluent production was high (Herrmann et al. 2015; Milledge and Harvey 2016a, b). As high initial %DM content reduces risks of environmental pollution in addition to improving silage stability, additional dewatering techniques are of interest to minimise seepage during the ensiling process and subsequent storage. Recently pre-ensiling treatments which increased macroalgal DM content by up to 16% were described by Gallagher et al. (2017). Working with one species of kelp, *Laminaria digitata* (Hudson) JV Lamouroux, they concluded that the preferred ensiling pre-treatment depended on the date of harvest.

Although ensiling is an attractive methodology for the preservation of macroalgae, the extremely diverse nature of this group of plants with many differences in morphology (thin flimsy thalli through to relatively thick blades with consequent differences in the physical nature and robustness of the material) and metabolites (proteins, lipids and particularly carbohydrates) (Suutari et al. 2015) means that no single approach may be applicable. Dewatering effects, in particular, may depend on the species of seaweed treated. It has only proved possible to extract juice from *L. digitata* in a screw-press after the alginates derived from cell wall alginic acid have been hydrolysed and the stickiness of the material removed (Adams et al. 2017; Gallagher et al. 2017).

In the work reported here, the dewatering study of Gallagher et al. (2017) has been widened to include two more species of brown seaweed (both kelps) and a red seaweed to examine the effects of variation in physical form and carbohydrate complement. *Laminaria hyperborea* (Gunnerus) Foslie is similar to *L. digitata*, but has thicker, tougher blades. *Saccharina latissima* (Linnaeus) C.E. Lane, C. Mayes, Druehl & G.W. Saunders has less robust blades. In contrast, the red alga, *Palmaria palmata* (Linnaeus) Weber & Mohr, is distinctly different both morphologically and biochemically with more delicate, easily damaged fronds that contain high concentrations of galactose-based compounds rather than alginates, mannitol and laminarin, found in all brown seaweeds (Jard et al. 2013; Suutari et al. 2015). The dewatering treatments tested previously, which included drying, osmotic media and the application of both organic and mineral acids all followed by screw-pressing to extract juice, have been applied to these seaweeds and the data combined with the previous results for *L. digitata*. Seaweed material was again collected at different times of year to examine the effects of seasonal variation in composition (Adams et al. 2011b; Schmid et al. 2014).

## Materials and methods

### Macroalgal material

All seaweed was sourced from wild stock in the UK between July 2014 and November 2016 and collected from intertidal beaches during afternoon spring low tides. Three to four kilogram of blade material was cut from the stipe/holdfast on each occasion. Local collections were returned to the laboratory within 1 h. Seaweed from further afield was transported in a cold box with ice blocks. All collections were then stored in sealed buckets at 4 °C overnight. Samples of seawater were taken at the same time from beside the collection point and also stored at 4 °C. Initial macroalgal dry matter (DM) content (%) was determined by oven drying at 70 °C for 6–7 days.

*Laminaria digitata* had been obtained from rocky outcrops at Aberystwyth north beach (52.4222° N, 4.0869° W) in January, April, July and October (Gallagher et al. 2017). *Palmaria palmata* was collected from the same location in May and August but was not available here in February or November after autumn and winter storms, and these harvests had to be sourced elsewhere. Consequently, in February and November, *P. palmata* was collected from more sheltered bays on south Gower (Langland Bay, 51.5686° N, 4.0123° W and Bracelet Bay, 51.5656° N, 3.9770° W), 75 miles from Aberystwyth. *Saccharina latissima* was also obtained from Langland Bay in February, May, August and November. Collections were designated spring, summer, autumn or winter in accordance with the meteorological seasons (spring = 1 March–31 May; summer = 1 June–31 August; autumn = 1 September–31 November; winter = 1 December–28 February). Material was obtained within a 4–5-week window for each season as determined by the dates of low tides. *Laminaria hyperborea* is only accessible during the lowest tides of the year and was initially only collected in March and September from rocky outcrops at Aberporth Bay (52.1360° N, 4.5449° W), 34 miles from Aberystwyth. It later proved possible to obtain material from below the water line in June on a calm day, but this species could not be collected in December. The *L. hyperborea* collection times fell outside the periods when the other species were collected, but they were still designated with the relevant meteorological season when included in some statistical analyses.

### Dewatering treatments

The dewatering treatments, described by Gallagher et al. (2017) and summarised in Table 1, were set up in random order within three replicate blocks. Clean macroalgal blade material was blotted dry and treated for 24 h at room temperature on the laboratory bench. Sample size was approximately 50 g material for the brown kelp seaweeds, but this had to be reduced to around 40 g for *P. palmata* to ensure complete submergence in treatment solutions. After treatment the material was stored at 4 °C in grip-seal plastic bags before being passed through a screw-press (Green Star Vegetable Juicer GS-1000; Tribest Corporation).

**Table 1:** Treatments applied to 40–50 g algal material in 1 L polypropylene lidded beakers for 24 h at room temperature (as Gallagher et al. 2017)

Code	Treatment	Applied as	
AIR	Air drying		Loosely folded, no lid
SALT	Dry salting (NaCl)	10 g	Shaken evenly over alga
FORMATE	Dry ammonium formate crystals	5 g	Shaken evenly over alga
SEA	Seawater	450 mL	Alga immersed
SALINE	Saline solution (10%)	450 mL	Alga immersed
DI	Ultrapure water	450 mL	Alga immersed
FORM C	Concentrated formic acid (23.6 M)	2 mL	Evenly over algal surface
FORM S	Formic acid solution (1%)	450 mL	Alga immersed
PROP C	Concentrated propionic acid (13.3 M)	2 mL	Evenly over algal surface
CRIMP C	Concentrated Crimpstore silage additive	2 mL	Evenly over algal surface
HCl C	Concentrated hydrochloric acid (11.6 M)	2 mL	Evenly over algal surface
HCl S	Hydrochloric acid solution (1%)	450 mL	Alga immersed
PHOS C	Concentrated phosphoric acid (14.7 M)	2 mL	Evenly over algal surface
PHOS S	Phosphoric acid solution (1%)	450 mL	Alga immersed

Five traits that have been shown to define the effects of dewatering treatment and screw-pressing macroalgal material (Gallagher et al. 2017) were derived, and the results expressed per 50 g sample for all species to allow direct comparison. These traits are the changes in total (fresh) weight, dry weight and water content resulting from treatment, the juice produced by screw-pressing and the final DM content (%) of the pressed residue.

### Statistical analysis

Data were analysed by analysis of variance (ANOVA) with the standard menu-driven procedures within GenStat edition 13 (VSN International). For identification of main effects, *L. hyperborea* was only included in analyses without ‘season’ as a factor. The ‘season’ designation of *L. hyperborea* was only included for full interaction analysis by 3-way ANOVA to assess differences between means. The treatment replicates were used as blocs to account for temporal variation from sample processing time. Post hoc multiple comparison analysis was carried out with the Tukey test using 95% confidence limits. Correlations were calculated as the product moment correlation coefficient for pair-wise combinations.

## Results

### Dewatering treatment and season effects

**Fig. 1:**
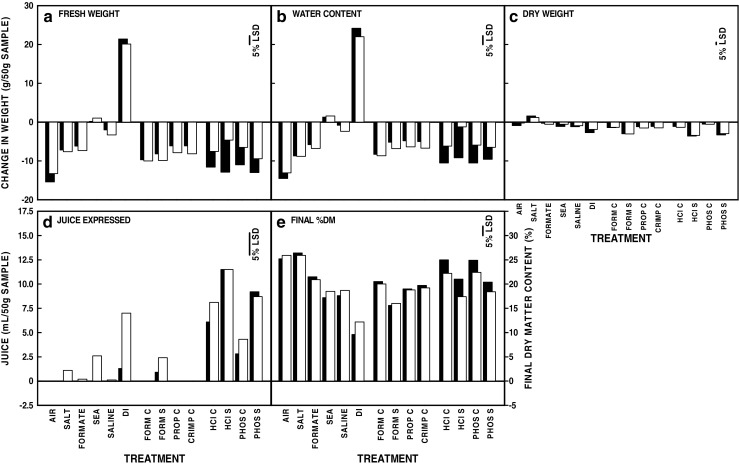
Effects of the application of different dewatering treatments to macroalgal samples for a period of 24 h. **a** Change in fresh weight (g (50 g)^−1^ material) from time zero (T0). **b** Change in water content (g (50 g)^−1^ material) from T0. **c** Change in dry weight (g (50 g)^−1^ material) from T0. **d** Juice produced by screw-pressing after treatment (mL (50 g)^−1^ g material). **e** Final dry matter content (%) following dewatering treatment and screw-pressing. Abbreviations for the different treatments are as shown in Table 1. Treatment means for all data for all the four macroalgal species (*n* = 45) are shown as open blocks. The least significant differences for comparisons at the 5% level are indicated. The *L. digitata* data from Gallagher et al. (2017) are shown as solid blocks in the background for information with no errors indicated

**Fig. 2:**
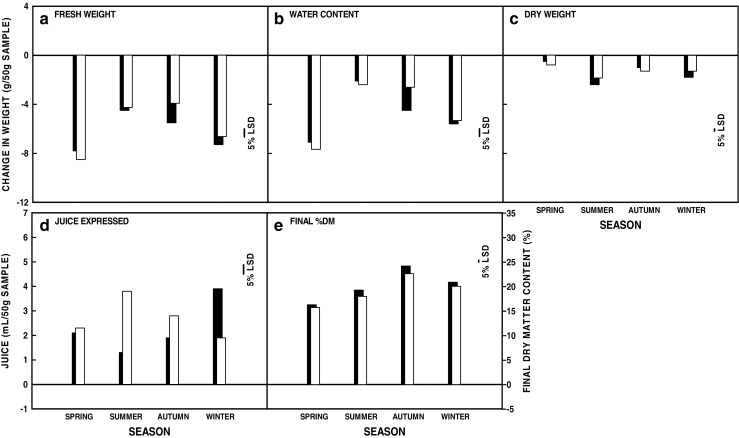
Seasonal variation in effects on macroalgal traits. **a** Change in fresh weight (g (50 g)^−1^ material) from time zero (T0). **b** Change in water content (g (50 g)^−1^ material) from T0. **c** Change in dry weight (g (50 g)^−1^ material) from T0. **d** Juice produced by screw-pressing after treatment (mL (50 g)^−1^ material). **e** Final dry matter content (%) following dewatering treatment and screw-pressing. Seasonal means for three species (*n* = 126—no *L. hyperborea*) are shown as open blocks. The least significant differences for comparisons at the 5% level are indicated. The *L. digitata* data from Gallagher et al. (2017) are shown as solid blocks in the background for information with no errors indicated

Main effect treatment means across all four species were similar to the previous data for *L. digitata* (Fig. 1). The only substantial differences observed were for the effects of the mineral acid treatments on fresh weight, water content and final biomass %DM content, and that juice was produced during screw-pressing following more of the treatments. In the case of seasonal means, juice extraction was also the only trait showing differences from *L. digitata* data with more juice produced in summer and less in winter (Fig. 2).

### Species variation

**Fig. 3:**
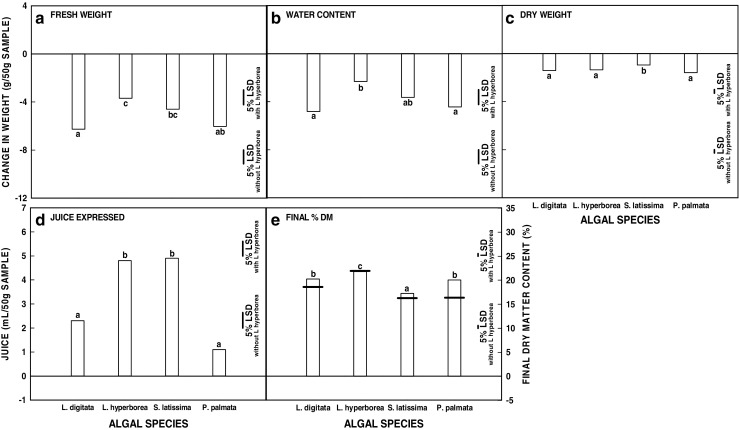
Effects of the application of dewatering treatments on different algal species. **a** Change in fresh weight (g (50 g)^−1^ material) from time zero (T0). **b** Change in water content (g (50 g)^−1^ material) from T0. c. Change in dry weight (g (50 g)^−1^ material) from T0. **d** Juice produced by screw-pressing after treatment (mL (50 g)^−1^ material). **e** Final dry matter content (%) following dewatering treatment and screw-pressing. Mean initial %DM at T0 is indicated by the horizontal line. Species means across all data (*n* = 168 except for *L. hyperborea* where *n* = 126). The least significant differences for comparisons at the 5% level with and without *L. hyperborea* are indicated. Blocks marked by the same letter are not significantly different at the 5% level as analysed by the Tukey multiple comparison test

**Fig. 4:**
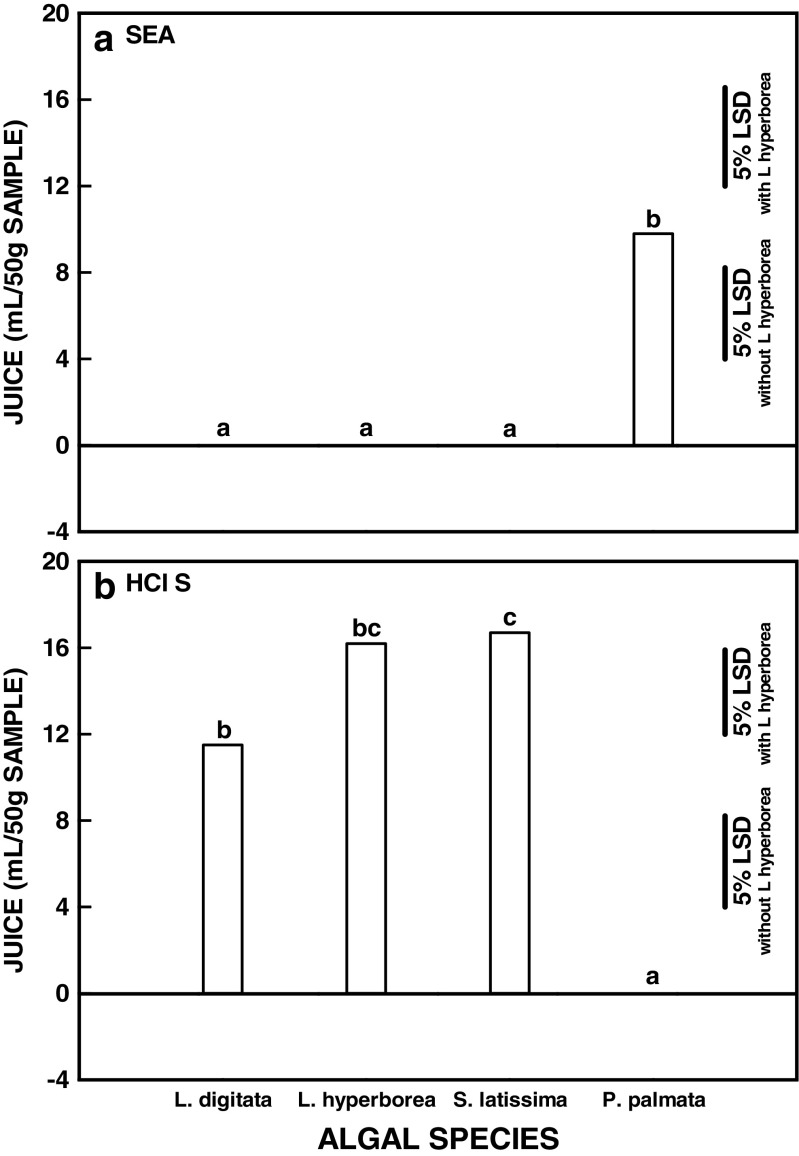
Effects of the application of two contrasting dewatering treatments on the behaviour of different algal species during screw-pressing. **a** Juice production (mL (50 g)^−1^ material) after treatment with seawater. **b** Juice production (mL (50 g)^−1^ material) after treatment with HCl solution. Species means across all seasons (*n* = 12 except for *L. hyperborea* where *n* = 9). The least significant differences for comparisons at the 5% level with and without *L. hyperborea* are indicated. Blocks marked by the same letter are not significantly different at the 5% level as analysed by the Tukey multiple comparison test

Species means across all treatments and harvest times are shown in Fig. 3. Despite their taxonomical, morphological and biochemical differences, *P. palmata* and *L. digitata* were not significantly different at the species main effect level for any of the traits of interest. *L. hyperborea* lost significantly less water and consequently less fresh weight during the dewatering treatments than *P. palmata*. Final %DM content was highest for *L. hyperborea* (although this was entirely a consequence of higher initial %DM) and lowest for *S. latissima*. *Saccharina latissima*, which had the lowest initial %DM content, lost least dry weight during dewatering treatment. More juice was extracted from *L. hyperborea* and *S. latissima* by screw-pressing. However, these main effect means obscure the extent of the species variation that was observed during the study as they represent the mean effect of all the dewatering treatments. The same mean can be produced when different treatments cause effects on the different seaweed species. For example juice production during screw-pressing after two treatments, the seawater control and 1% hydrochloric acid solution, is shown for the four species in Fig. 4. *Palmaria palmata* was the only species from which juice could be extracted after the seawater treatment (Fig. 4a) whereas only *L. digitata*, *L. hyperborea* and *S. latissima* produced juice on pressing after acid treatment (Fig. 4b). In fact, acid treatment was too severe for the more delicate physical nature of the *P. palmata* fronds and led to loss of the structural morphology of the biomass. The significance levels from three-way ANOVA of algal species, treatment and season across the study were highly significant (*P* < 0.001) for all interactions as well as all main effects (Table 2) showing that the effect of treatment varied significantly between the algal species and that this was further modified by the time of year the seaweeds were collected.

**Table 2:** Significance levels from two separate ANOVA analyses. Three-way analysis for the effects of alga, treatment, season and their interactions was carried out with data for *L. digitata*, *S. latissima* and *P. palmata*. Results from two-way analysis without season effects carried out with data for all four species are shown in the lower section of the split cells

	Change in fresh weight (g (50 g)^−1^ sample)	Change in water content (g (50 g)^−1^ sample)	Change in dry weight (g (50 g)^−1^ sample)	Juice expressed (mL (50 g)^−1^ sample)	Final DM content (%)
Alga	*P* < 0.001	*P* < 0.001	*P* < 0.001	*P* < 0.001	*P* < 0.001
	*P* < 0.001	*P* < 0.001	*P* < 0.001	*P* < 0.001	*P* < 0.001
Treatment	*P* < 0.001	*P* < 0.001	*P* < 0.001	*P* < 0.001	*P* < 0.001
	*P* < 0.001	*P* < 0.001	*P* < 0.001	*P* < 0.001	*P* < 0.001
Season	*P* < 0.001	*P* < 0.001	*P* < 0.001	*P* < 0.001	*P* < 0.001
Alga.Treatment	*P* < 0.001	*P* < 0.001	*P* < 0.001	*P* < 0.001	*P* < 0.001
	*P* < 0.001	*P* < 0.001	*P* < 0.001	*P* < 0.001	*P* < 0.001
Alga.Season	*P* < 0.001	*P* < 0.001	*P* < 0.001	*P* < 0.001	*P* < 0.001
Treatment.Season	*P* < 0.001	*P* < 0.001	*P* < 0.001	*P* < 0.001	*P* < 0.001
Alga.Treatment.Season	*P* < 0.001	*P* < 0.001	*P* < 0.001	*P* < 0.001	*P* < 0.001

### Variation in final %DM content

The traits with most impact on processing considerations are final %DM content and juice extraction by screw-pressing as the aim of dewatering treatment was to increase DM content for better ensiling. Seventeen of the 20 species by treatment by season combinations with the highest final %DM content were for material obtained in autumn when algal %DM content was highest. Half these 20 combinations were for *L. hyperborea*, but this was as much a consequence of high initial %DM content as of an increase in %DM content resulting from dewatering treatment. For example, the mean initial %DM content of *L. hyperborea* collected in September (autumn) was 31.7%. The top performing treatments for each season and species are shown on Table 3. Data for dry salt and formate treatments were not included in the ANOVA as they showed anomalous weight increases. The highest %DM contents were predominantly for air drying or the concentrated acid treatments. Air drying *L. hyperborea* in autumn resulted in the highest %DM content observed in the study (Table 3 (A)). Air-drying *P. palmata* and immersing *L. digitata* in hydrochloric acid solution were most effective for material obtained in winter (Table 3 (B)). Across the full dataset (but without dry salt, dry formate and the DI treatments), there was a low but highly significant negative correlation (correlation coefficient − 0.2638; *n* = 486, *P* < 0.001) between the magnitude of dry weight loss and %DM content: that is, the greater the dry weight lost during pre-treatment the lower the final %DM content of the algal material. A similar correlation was also found within the data subset of the three treatments leading to the highest final %DM contents (air and concentrated mineral acid (Table 3); correlation coefficient − 0.3650; *n* = 129, *P* < 0.001).

**Table 3:** Interaction level means (*n* = 3) for final %DM content of the top two treatments for each season and species. Data in the top section of the table (A) are from ANOVA with all four species and three seasons. Data for winter below (B) are from ANOVA with three species (without *L. hyperborea*) and all four seasons. The combined standard deviation of the mean from ANOVA was 2.099 for section (A) and 1.521 for section (B). Means within each section followed by the same letter (lower and upper case are different) are not significantly different at the 5% level according to the Tukey multiple comparison test

Season	Algal species	Treatment	Final %DM content
A			
Spring	*L. digitata*	PHOS C	20.80 klmnopqrstuvwxyzABCDEFGHI
Spring	*L. digitata*	HCl C	20.10 opqrstuvwxyzABCDEFGHIJKLMN
Spring	*L. hyperborea*	AIR	32.24 abc
Spring	*L. hyperborea*	FORM C	26.16 bcdefghijklmnopqr
Spring	*S. latissima*	PHOS C	20.34 nopqrstuvwxyzABCDEFGHIJKL
Spring	*S. latissima*	HCl C	17.56 tuvwxyzABCDEFGHIJKLMNOPQRSTU
Spring	*P. palmata*	AIR	23.97 efghijklmnopqrstuvwx
Spring	*P. palmata*	SEA	19.33 qrstuvwxyzABCDEFGHIJKLMNOP
Summer	*L. digitata*	SEA	25.58 bcdefghij
Summer	*L. digitata*	PHOS C	25.07 cdefghijklmnopqrstu
Summer	*L. hyperborea*	AIR	18.96 qrstuvwxyzABCDEFGHIJKLMNOPQ
Summer	*L. hyperborea*	FORM C	17.90 tuvwxyzABCDEFGHIJKLMNOPQRSTU
Summer	*S. latissima*	HCl C	22.20 ijklmnopqrstuvwxyzAB
Summer	*S. latissima*	PHOS C	20.72 lmnopqrstuvwxyzABCDEFGHIJ
Summer	*P. palmata*	AIR	28.54 bcdefghijk
Summer	*P. palmata*	SEA	28.17 bcdefghijklm
Autumn	*L. digitata*	HCl C	30.34 bcdefg
Autumn	*L. digitata*	AIR	30.13 bcdefgh
Autumn	*L. hyperborea*	AIR	38.54 a
Autumn	*L. hyperborea*	HCl C	33.86 ab
Autumn	*S. latissima*	AIR	27.87 bcdefghijklmn
Autumn	*S. latissima*	HCl C	27.47 bcdefghijklmno
Autumn	*P. palmata*	AIR	31.96 abcd
Autumn	*P. palmata*	SEA	24.46 defghijklmnopqrstuvw
B			
Winter	*L. digitata*	HCl S	31.29 a
Winter	*L. digitata*	HCl C	26.55 abcdefghi
Winter	*S. latissima*	HCl C	23.75 cdefghijklmno
Winter	*S. latissima*	PHOS C	23.46 defghijklmnopqr
Winter	*P. palmata*	AIR	31.70 a
Winter	*P. palmata*	DI	23.63 cdefghijklmnopq

### Variation in juice extraction by screw-pressing

Juicing can sometimes be used to increase %DM content of biomass and at the same time provide a useful liquid by-product. The 20 species by treatment by season combinations with the highest yield of juice from screw-pressing showed less seasonal bias than the final %DM content data. Over half these combinations were for *S. latissima* samples which produced significant volumes of juice across a range of treatments at different times of year. The top performing treatments for each season and species are shown on Table 4. Data for dry salt and formate treatments were again excluded, as was the DI treatment since this never yielded more juice than the volume of water absorbed during the 24 h treatment. Nearly three quarters of these combinations included acid treatment, particularly with mineral acid solutions. The data indicate that it is likely that screw-pressing kelp after treatment with mineral acid solutions significantly increased final %DM content. Within the data subset for the three kelp species following treatment with hydrochloric and phosphoric acid solutions, there was a low but significant correlation (correlation coefficient 0.2309; *n* = 66, *P* < 0.05) between the volume of juice produced and %DM content. In contrast *P. palmata* always produced most juice after immersion in seawater (Table 4). In fact, screw-pressing algal biomass of *P. palmata* collected in summer and subjected to the seawater control treatment for 24 h resulted in the highest volume of juice observed in this study (Table 4 (A)) and also produced dry residue with 28.17% DM content (Table 3 (A)). This raised the possibility that *P. palmata* would press as fresh, untreated material directly after collection. This was tested at laboratory bench scale with material from a May collection. Screw-pressing 433 g material that had been briefly rinsed in tap water and drained produced 108 mL juice and 185 g residue. The %DM content significantly increased from 10.9 ± 0.2 for the fresh material to 14.1 ± 0.4 for the screw-press residue.

**Table 4:** Interaction level means (*n* = 3) for the top two treatments for volume of juice extracted for each season and species. Data in the top section of the table (A) are from ANOVA with all four species and three seasons. Data for winter below (B) are from ANOVA with three species (without *L. hyperborea*) and all four seasons. The combined standard deviation of the mean from ANOVA was 5.01 for section (A) and 3.97 for section (B). Means within each section followed by the same letter are not significantly different at the 5% level according to the Tukey multiple comparison test

Season	Algal species	Treatment	Juice (mL)
A			
Spring	*L. digitata*	HCl S	12.2 fghijk
Spring	*L. digitata*	PHOS S	9.4 ijklmnop
Spring	*L. hyperborea*	HCl S	12.4 fghijk
Spring	*L. hyperborea*	HCl C	11.4 ghijklm
Spring	*S. latissima*	HCl S	17.6 abcdefg
Spring	*S. latissima*	HCl C	17.5 abcdefg
Spring	*P. palmata*	SEA	5.1 mnopqrst
Spring	*P. palmata*	HCl C	0.8 qrstu
Summer	*L. digitata*	PHOS S	5.3 mnopqrst
Summer	*L. digitata*	HCl S	4.6 nopqrstu
Summer	*L. hyperborea*	HCl S	17.7 abcdefg
Summer	*L. hyperborea*	HCl C	13.4 defghij
Summer	*S. latissima*	HCl C	22.7 ab
Summer	*S. latissima*	PHOS S	20.6 abc
Summer	*P. palmata*	SEA	24.1 a
Summer	*P. palmata*	HCl S	0.0 stu
Autumn	*L. digitata*	HCl S	10.2 hijklmno
Autumn	*L. digitata*	PHOS S	9.5 ijklmnop
Autumn	*L. hyperborea*	HCl S	18.5 abcdef
Autumn	*L. hyperborea*	PHOS S	12.5 efghijk
Autumn	*S. latissima*	HCl S	19.6 abcd
Autumn	*S. latissima*	PHOS S	19.3 abcd
Autumn	*P. palmata*	SEA	6.4 klmnopqrs
Autumn	*P. palmata*	SALINE	0.8 qrstu
B			
Winter	*L. digitata*	HCl S	18.9 abc
Winter	*L. digitata*	HCl C	12.9 def
Winter	*S. latissima*	HCl S	10.6 fghi
Winter	*S. latissima*	PHOS S	4.7 klmnopq
Winter	*P. palmata*	SEA	3.5 lmnopq
Winter	*P. palmata*	PHOS C	0.0 q

## Discussion

### Macroalgal biomass as feedstock for sustainable fuel production

Macroalgal biomass may be suitable as direct feedstock for some cascading-extraction biorefinery processes for the isolation of high value components if these processes happen locally and transportation costs are therefore not an issue. However in the case of the production of sustainable fuels, further considerations apply. First and second generation biofuels were derived from ‘dry’ biomass. Third generation fuels are produced from microalgae and seaweeds which are ‘wet’ biomass (Anastasakis and Ross 2015). Processes which avoid the need for any dehydration step by directly using wet feedstock are therefore quite competitive (Chen et al. 2015; Song et al. 2015; Jiang et al. 2016; Shobana et al. 2017). However, although fermentation (for biohydrogen) and anaerobic digestion (for methane) are suitable for wet biomass, they are not necessarily the best approach to take with seaweeds due to the metabolite composition of these materials (Anastasakis and Ross 2015) and many macroalgae may be more suited to thermochemical conversions (Choi et al. 2014). Consequently, there is increasing interest in developing seaweed-based combustion, pyrolysis, and hydrothermal liquefaction and gasification processes for sustainable fuel production (Anastasakis and Ross 2015). The drawback is the requirement in many cases for dry feedstock. For example pyrolysis, where, largely due to energy requirements as high as 63% of total process for drying wet biomass (Seghetta et al. 2016), life cycle analyses have shown production (financial and energy) costs to be greater than product (fuel) value (Jiang et al. 2016). Although poor, this may be more acceptable for some particular fuel applications such as aviation fuel, where other forms of renewable energy are currently not feasible (Raven 2017).

### Dewatering algae

The same considerations to reduce dewatering costs to improve sustainability apply equally to microalgae and macroalgae (Choi et al. 2014; Sahoo et al. 2017; Shastri 2017). Freeze-drying, spray-drying and oven-drying are all highly energy intensive (Sahoo et al. 2017). However, whilst dewatering techniques for microalgae have been widely studied and best available technologies include spiral plate centrifuging, heat-assisted rotary pressure filtering, heat-integrated drying and forward osmosis employing proton exchange membranes (Seghetta et al. 2017; Son et al. 2017), research with macroalgae is comparatively much less advanced.

Experimentally most workers have dried macroalgae in an oven before further processing although sun-drying, freeze-drying and microwave-drying have all been employed, particularly before fermentations (Adams et al. 2015, 2017) and pyrolysis (Zhao et al. 2013; Choi et al. 2014; Balboa et al. 2015; Roberts et al. 2015; Kostas et al. 2017). Different drying methods show differences in maintenance of biomass quality, with freeze-drying best for metabolite stability and sun-drying worst due to the long time frame (Chan et al. 1997). However, the energy costs of mechanical dewatering are typically an order of magnitude lower than for thermal drying because no phase change of water is involved (Lightfoot and Raghavan 1994), so it is clear that the cost (both in financial and energy terms) of processing kelp could be significantly reduced by mechanical dewatering. Unfortunately currently established methodologies have not always been able to deliver sufficiently low water contents to realise these significant advantages over thermal methods (Mahmoud et al. 2010). The small number of studies that have been published has shown that kelp is difficult to dewater because its slippery gel-like nature strongly binds water (Lightfoot and Raghavan 1994; Gallagher et al. 2017). Both reports showed < 1% water was removed by screw-pressing. Adams et al. (2017) tried including a press aid during screw-pressing but still assessed that < 10% material (both water and particulates) could be separated.

Further research on mechanical dewatering methods for brown seaweeds to match progress in microalgal research is urgently required in order to fully exploit this valuable biomass resource. One promising technique is combined fields dewatering (e.g., mechanical pressure and electro-osmosis) which gave improvement over conventional press dewatering and has been successfully applied to kelp suspensions (Lightfoot and Raghavan 1994; Orsat et al. 1999; Mahmoud et al. 2010).

### Macroalgal pre-treatment

Another approach is to identify pre-treatments for kelp that increase its amenability to established mechanical dewatering technology such as various types of pressing. In this study, *L. hyperborea* and *S. latissima* could both be dewatered and the main treatment and season effects were very similar to those previously determined for *L. digitata* (Gallagher et al. 2017). Previously, it was shown that *L. digitata* produced most juice (and biomass with high %DM content) when collected in winter and subjected to treatment with 1% hydrochloric acid for 24 h (Gallagher et al. 2017). Like *L. digitata*, *L. hyperborea* required mineral acid treatment before juice was produced during screw-pressing. *Saccharina latissima* produced juice following a wider range of treatments (mainly acid-based but including organic as well as mineral acids) although this did not always lead to material with significantly increased %DM content. Other workers have found acids to be useful pre-treatments. Hydrochloric acid solution, followed by hot-water blanching and belt-platen pressing, was shown to decrease kelp water content by around 50% (Lightfoot and Raghavan 1994). Hart et al. (1978) achieved a 75% moisture reduction after treatment with calcium chloride, heat and pressing.

More generally such treatments can also be part of sequential extraction protocols and/or advantageous in respect of biomass properties during downstream processing in cascading biorefinery systems which aim to maximise the inherent value of all components present in biomass (Kostas et al. 2017). Acid treatments (Chen et al. 2015; Jiang et al. 2016; Kostas et al. 2017) and sometimes water washes (Choi et al. 2017) feature most prominently in the literature on sustainable fuel production from macroalgae. Acid treatments are effective initial extraction steps for valuable carbohydrates (Lorbeer et al. 2015; Ryu and Keun 2017) and have been shown to improve both feedstock fermentability (Ryu and Keun 2017) and the operation of continuous pyrolysis systems without affecting the quality of the pyrolysis oils produced (Choi et al. 2014). The acidification resulting from ensiling can have a similar effect on all impact categories of life cycle analysis (Seghetta et al. 2017). While whole seaweed extracts and powders made from them have been widely used in organic farming and horticulture as biofertilisers and as biostimulants to promote improved seedling survival, plant growth and crop yields (MacMonagail et al. 2017), the final mineral residues from such biorefinery and sustainable fuel applications can also be useful as fertilisers and for soil amelioration (Kraan 2013; Roberts et al. 2015; Manns et al. 2017).

Therefore, in general, mineral acid treatments have been proved suitable and effective for dewatering and pressing brown alginate-containing seaweeds. This study though, does suggest that further treatment before ensiling for algae like *L. hyperborea* with high DM content across much of the year may be less important than for other species. However, pre-treatment before mechanical pressing is not appropriate or necessary for all seaweeds. In this study, acid treatments in particular were not at all suitable for the red alga *P. palmata*. These were too severe for the physical nature of the biomass and did not lead to juice production in the screw-press or material with increased %DM content but rather to loss of the structural morphology of the biomass resulting in an amorphous mass which retained water. *Palmaria palmata* could, though, be screw-pressed immediately after collection which resulted in high juice yields and increased biomass %DM content.

### Carbohydrates and seasonal variation

Different outcomes were observed when the acid treatment was applied as concentrated acid or as a dilute solution. Concentrated acids were better at producing material with high final %DM content but not material that would screw-press. Dilute acid solutions were more effective at reducing stickiness and producing material that would produce juice in a screw-press. The difficulties with mechanical dewatering of kelp have been attributed to the presence of alginates and their hydrocolloidal properties (Lightfoot and Raghavan 1994). Certainly in this study, *S. latissima*, which was noticeably less sticky than the *Laminaria* species on most occasions and is reported to have lower alginate content (Schiener et al. 2015), produced juice following a wider range of treatments. It seems likely that immersion in solutions was more effective at hydrolysing and ‘washing out’ the alginate. Carbohydrate composition of kelp varies throughout the year, with maximum ash, protein, and matrix polysaccharides (alginate, fucoidan) contents at the beginning of the spring, when the reserve compounds mannitol and laminarin are at a minimum (Adams et al. 2011b; Manns et al. 2014; Schmid et al. 2014). Glucan levels were highest in late summer and autumn with the highest %DM also occurring at this time (Manns et al. 2017). As a consequence of this seasonal variation in %DM content, water content in seaweeds (like all photosynthetic plants) therefore also shows seasonal variation as these measures are effectively reciprocal proportions of total weight. In this study, water content and %DM did indeed vary in this manner. Acid treatments were observed to be effective across the year perhaps because alginate content has been reported to vary less than reserve carbohydrate content (Manns et al. 2017). It follows that water holding capacity may therefore show less seasonal variation than water content.

### Species choice

In this study, there were significant differences between the algae for all the traits of interest and numerous specific interactions between species, treatment and season which could be used to inform individual bioprocessing trials. However, although knowledge of species variation in dewatering and preservation of macroalgal material will be critical for planning seaweed-based processing streams, this will be irrelevant without a suitable supply of biomass. Critical decisions in offshore biomass production for biofuels relate to species choice, productivity, environmental conditions and application economics plus any potential role in bioremediation (Fernand et al. 2017). Species selection will need to be as much about biomass production as about biomass processing.

In recent years, the European Union has invested millions in research projects to support macroalgae aquaculture, mainly of kelps, to boost biomass production for bio-derived fuel manufacturing purposes (e.g. EnAlgae, MABFUEL, AT-SEA). Several species including *Laminaria* sp., *S. latissima* and *Alaria esculenta* have been trialled and successfully farmed on ropes across the species latitude range from Northern to Southern Europe (Norway to Portugal) (Arbona and Molla 2006; Edwards and Watson 2011). The success of this approach is reflected in worldwide annual yields of brown seaweeds from rope cultivation reported to be between 12 and 60 t dry matter ha^−1^ year^−1^ (Bruton et al. 2009). The life cycle, cultivation techniques and yield of these kelp species are similar—they reproduce through spores, which upon germination grow directly into gametophytes that produce gametes. Once fertilization occurs the resulting zygote grows into a sporophyte thus completing the sexual life cycle. Kelp sporophytes develop on ropes or strings at sea after direct settling of spores, gametophytes or sporophytes in controlled conditions in seaweed hatcheries (Arbona and Molla 2006; Edwards and Watson 2011). *Palmaria palmata* has also been trialled at sea using ropes and nets and in tanks, but it is less economically feasible to farm than the kelps (Wemer and Dring 2011). The *P. palmata* yield reported by Wemer and Dring (2011) was 750 g per linear metre of culture string after about 5 months. For all species, site selection, time of deployment and duration of cultivation is crucial. Some sites may be ideal for farming kelp species, but inappropriate for growing *P. palmata* (Wemer and Dring 2011). Suitable currents, good water exchange and sea temperature are key environmental factors for site selection and vary depending on the species. In general, the species studied are deployed in late autumn/early winter and harvested after 5 months in late spring/early summer.

## Conclusions

In conclusion, sufficient information is now available to plan scaled-up dewatering and ensiling trials in some cases, for example with screw-pressed fresh *P. palmata* harvested in the summer or *L. digitata* obtained in winter, treated with hydrochloric acid solution and screw-pressed. However it appears there may be no universal pre-treatment for optimal dewatering of macroalgae in preparation for ensiling. Overall, the effects of dewatering treatments, including drying, osmotic media and the application of both organic and mineral acids all followed by screw-pressing, were found to be highly dependent on algal species, season of collection and treatment. In general, it will be necessary to carry out preliminary tests on new candidate species to establish best processing protocols. Species selection will need to be considered on a case-by-case basis in a complex matrix, which includes not only ease of dewatering and suitability for ensiling but also elements of macroalgal culture and potential biomass supply. Individual species ecology, physiological needs and site selection logistics including ease of access from shore, distance from the shore, licensing difficulties and user conflicts will all be important.
